# Growth response of syndromic versus non-syndromic children born small for gestational age (SGA) to growth hormone therapy: a Belgian study

**DOI:** 10.3389/fendo.2023.1112938

**Published:** 2023-06-02

**Authors:** Marianne Becker, Muriel Thomas, Cécile Brachet, Claudine Heinrichs, Hilde Dotremont, Jean De Schepper, Philippe Lysy, Dominique Beckers, the BESPEED group, Anne Rochtus

**Affiliations:** ^1^ Department of Pediatric Endocrinology and Diabetology, Centre Hospitalier de Luxembourg, Luxembourg, Luxembourg; ^2^ The BElgian Society for PEdiatric Endocrinology and Diabetology (BESPEED), Brussels, Belgium; ^3^ Pediatric Endocrinology Unit, Hôpital Universitaire des Enfants Reine Fabiola (HUDERF), Université libre de Bruxelles (ULB), Brussels, Belgium; ^4^ Department of Pediatric Endocrinology and Diabetology, University Hospital Antwerp, Edegem, Belgium; ^5^ University Hospital of Brussels, Brussels, Belgium; ^6^ Department of Pediatric Endocrinology and Diabetology, UCLouvain, Brussels, Belgium; ^7^ Department of Pediatric Endocrinology and Diabetology, UCLouvain, CHU UCL Namur, Yvoir, Belgium

**Keywords:** short for gestational age, syndromic, growth hormone, growth, children, adult height, short stature

## Abstract

**Introduction:**

A substantial proportion of SGA patients present with a syndrome underlying their growth restriction. Most SGA cohorts comprise both syndromic and non-syndromic patients impeding delineation of the recombinant human growth hormone (rhGH) response. We present a detailed characterization of a SGA cohort and analyze rhGH response based on adult height (AH).

**Methods:**

Clinical and auxological data of SGA patients treated with rhGH, who had reached AH, were retrieved from BELGROW, a national database of all rhGH treated patients held by BESPEED (BElgian Society for PEdiatric Endocrinology and Diabetology). SGA patients were categorized in syndromic or non-syndromic patients.

**Results:**

272 patients were included, 42 classified as syndromic (most frequent diagnosis (n=6): fetal alcohol syndrome and Silver-Russell syndrome). Compared with non-syndromic patients, syndromic were younger [years (median (P10/P90)] 7.43 (4.3/12.37) vs 10.21 (5.43/14.03), p=0.0005), shorter (height SDS -3.39 (-5.6/-2.62) vs -3.07 (-3.74/-2.62), p=0.0253) and thinner (BMI -1.70 (-3.67/0.04) vs -1.14 (-2.47/0.27) SDS, p=0.0054) at start of rhGH treatment. First year rhGH response was comparable (delta height SDS +0.54 (0.24/0.94) vs +0.56 (0.26/0.92), p=0.94). Growth pattern differed with syndromic patients having a higher prepubertal (SDS +1.26 vs +0.83, p=0.0048), but a lower pubertal height gain compared to the non-syndromic group (SDS -0.28 vs 0.44, p=0.0001). Mean rhGH dose was higher in syndromic SGA patients (mg/kg body weight/day 0.047 (0.039/0.064) vs 0.043 (0.035/0.056), p=0.0042). AH SDS was lower in syndromic SGA patients (-2.59 (-4.99/-1.57) vs -2.32 (-3.3/-1.2), p=0.0107). The majority in both groups remained short (<-2 SDS: syndromic 71%, non-syndromic 63%). Total height gain was comparable in both groups (delta height SDS +0.76 (-0.70/1.48) vs +0.86 (-0.12/1.86), p=0.41).

**Conclusions:**

Compared to non-syndromic SGA patients, syndromic SGA patients were shorter when starting rhGH therapy, started rhGH therapy earlier, and received a higher dose of rhGH. At AH, syndromic SGA patients were shorter than non-syndromic ones, but their height gain under rhGH therapy was comparable.

## 1 Introduction

Three percent of all children are born small for gestational age (SGA), of those 10-13% do not develop catch-up growth and remain short (1–3). Treatment with recombinant human growth hormone (rhGH) was reported to increase significantly adult height (AH) in short children born SGA (4–7). Based on these results, the European Medicines Agency (EMA) approved in 2003 rhGH therapy for children born SGA who are lacking catch-up growth at the age of 4 years.

Nonetheless, the response to growth hormone therapy is very variable and several studies have been published, trying to identify predicting factors for growth response in SGA patients (8, 9). One of the discussed reasons for the variable growth response, is that SGA patients are a heterogenous group including patients who suffered from an intrauterine growth restriction caused by a variety of reasons: maternal complication (preeclampsia, uterine anomalies, maternal drug use, including alcohol and tobacco), fetal complications (intrauterine infections, syndromes), placental abnormalities (reduced placental blood flow) and environmental insults (toxic substances, altitude) (10).

The reported cohorts often contain patients suffering from a severe or partial growth hormone deficiency (4, 5, 11), or patients who were additionally treated with GnRH analogues (12), which might further influence the variable growth hormone response of the studied cohort (13).

Syndromic patients are reported to respond worse to rhGH therapy than non-syndromic patients (12). In some studies, syndromic patients have been excluded (11, 14), while in others, only Silver-Russell Syndrome (SRS) patients were included (6, 15). Dahlgren et al. excluded SGA patients with chromosomal disorders, chondrodysplasia, fetal alcohol syndrome (FAS) and children with “serious malformations”, but included a SRS patient (5). So, the published SGA cohorts are very heterogenous. A recent study examined rhGH response during the first two years of therapy in the following SGA subgroups: patients with dysmorphic features, FAS patients and SRS patients. This study revealed the best response to rhGH in the SRS subgroup and the highest rate of non-responders, defined as delta height SDS <0.3 after the first year of rhGH therapy, in the FAS subgroup (16). Data on rhGH response for syndromic patients on adult height are sparse. Few studies have published adult height after growth hormone therapy for SRS patients (17–19). They show a lower adult height, but an equal height gain compared to non-SRS SGA patients under rhGH therapy (18, 19).

We report on a large SGA cohort retrieved from the Belgian national registry for patients treated with growth hormone (BELGROW) held by BESPEED (BElgian Society for PEdiatric Endocrinology and Diabetology) and determined how many syndromic patients were included in this cohort and which syndromes had been diagnosed. We further compared the two SGA groups (syndromic versus non-syndromic SGA) and analysed their response to rhGH therapy.

## 2 Material and methods

### 2.1 Subjects

**Figure 1:**
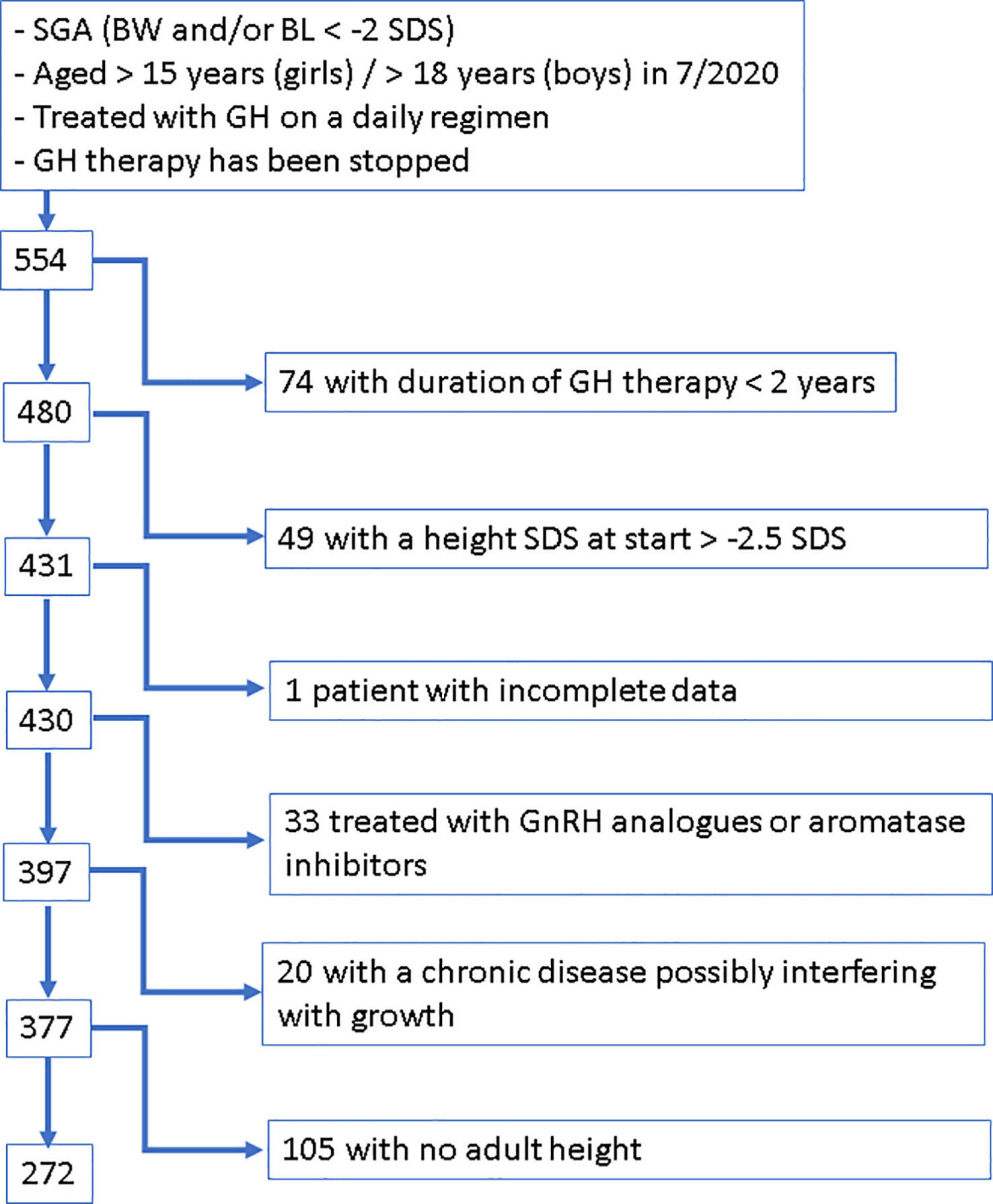
Flow Chart visualizing the applied procedure of patient selection.

SGA patients who fulfilled the following criteria were extracted from BELGROW: birth length and/or birth weight <-2 SDS according to Niklasson (20) and for children born preterm <28.5 weeks of gestation according to Intergrowth (21, 22), who had a height <-2.5 SDS according to Roelants (23) when starting rhGH therapy, who were treated at least 2 years with daily subcutaneous rhGH injections and for whom an AH was documented in BELGROW (**Figure 1**).

Patients were excluded if they had been treated with aromatase inhibitors or GnRH analogues, if they were suffering from a chronic disease known to possibly interfere with growth, such as chronic intestinal diseases, cystic fibrosis, cardiac insufficiency, precocious puberty, 21 hydroxylase deficiency, immune deficiency syndromes, oncological disease, severe hypothyroidism and spastic paralysis, if they were diagnosed with or had symptoms of bone dysplasia, or if they had a genetically confirmed mutation in the IGF-1 receptor gene.

After applying these criteria, our SGA cohort included 272 patients.

These patients were categorized into syndromic and non-syndromic patients. All patients who had a genetically confirmed syndrome, or a syndrome diagnosis based on a published clinical score (SRS, FAS) or who had in addition to their short stature at least two other symptoms (congenital heart defects, intellectual disability, dysmorphic features, …) suggesting a syndromic origin of their short stature were classified as syndromic SGA patients.

BELGROW is a database, which has been running since 1985 by BESPEED and includes almost all patients treated with rhGH in Belgium. This registry stores pseudonymized data. Informed consent of the registered patients has been obtained.

### 2.2 Methods

Variables retrieved from BELGROW were: diagnosis; gender; weight and length at birth; father’s and mother’s height; age, height, weight, pubertal stage at start of rhGH therapy, after 1 year, at start of puberty, at end of rhGH therapy and at adult height.

SDS calculations were performed applying reference values published by Niklasson (20) for birth parameters and Intergrowth data (21, 22) for preterm babies <28.5 weeks of gestation. For follow-up data we used reference data published by Roelants (23) to determine SDS.

AH was assumed if growth velocity was less than 2 cm/year and pubertal development was completed (Tanner stage 5 and/or min. 2 years after menarche in girls; min. testicular volume of 15 ml in boys) and/or bone age or estimated bone age (24) was min. 14 years in girls or min. 16 years in boys. As our applied growth velocity to define adult height was not 0 cm/year and as we expressed AH in SDS by applying the gender-adapted SDS of the age of 21 to the obtained AH, we are certainly underestimating adult height slightly.

Mean daily dose (mg/kg body weight/day) during the first year and during the whole treatment period was calculated using the dosage recorded at each visit.

Mid-parental height (MPH) was calculated by [father’s height (cm) + mother’s height (cm) + 13 cm for boys/- 13 cm for girls]/2 (25). Target height range was defined as MPH +/- 10 cm for boys and MPH +/- 9 cm for girls (26).

The response of growth hormone therapy was evaluated by the change from baseline height standard deviation score to AH standard deviation score (Δ-height SDS).

### 2.3 Statistical analysis

Results are expressed as median (P10-P90) or percentages. The percentage of subjects with an AH SDS <-2 and with an AH in their target height range was calculated. Continuous variables and percentages were compared across groups using Mann-Whitney U tests, or chi-square tests as appropriate. A p value <0.05 was considered statistically significant. Stata 15.1 was used for statistical analysis.

## 3 Results

### 3.1 Cohort characterization

We identified 42 syndromic patients in our SGA cohort (15%).

The most frequent diagnosis was FAS (six), and SRS (six), followed by patients with 3M syndrome (two). The diagnosis of a defined syndrome was mostly made before the start of treatment. Twenty-five of the 42 syndromic patients had a defined syndrome, hence 17 (40%) had no defined syndrome (**Table 1**). Genetic analysis in 8 of these 17 patients was not contributory. Genetic analysis included karytoype, CGH-array, specific gene analysis and whole exome sequencing. These methods were used either individually or in different combinations with or without a genetic consultation. As our data are based on a registry, which is used by different physicians from different Belgian centers, there was no uniform approach for the genetic work-up.

**Table 1:** Description of the syndromic SGA group.

Syndromes	N =42
Silver-Russell syndrome	6
Fetal alcohol syndrome	6
3M syndrome	2
Becker dystrophia	1
Di George syndrome	1
Klinefelter	1
Mulvihill -Smith syndrome	1
Ohdo Blepharophimosis syndrome	1
Pierre Robin Sequence	1
Ring chromosome 11	1
Ring chromosome 7 in mosaicism	1
Renpenning syndrome	1
VATER syndrome	1
Seckel syndrome	1
Non defined syndromes	17

In 9 patients, no genetic analysis had been performed.

### 3.2 Syndromic versus non-syndromic SGA patients: Comparison of baseline characteristics

In the syndromic group there was a higher percentage of male patients (71%) compared to the non-syndromic group (55%), but this was not statistically significant (p=0.051). Gestational age did not differ between the two groups, but syndromic patients had lower birth weight (-2.83 versus -2.26 SDS, p=0.0011) and length (-3.09 versus -2.48 SDS, p=0.0178) than non-syndromic patients at birth (**Table 2A**).

Table 2Syndromic versus non-syndromic SGA patients: Comparison of baseline characteristics.

**Table:** 

A: Syndromic versus non-syndromic SGA patients: Birth parameters and parental heights							
	Syndromic SGA			Non-Syndromic SGA			Comparison of the 2 groups
	n=42			n=230			
	median or n (%)	P10	P90	median or n (%)	P10	P90	p
Gender Male/Female	**30 (71%)/12 (29%)**			**127 (55%) /103 (45%)**			p=0.051
Birth weight SDS	**-2.83**	-4.36	-1.34	**-2.26**	-3.44	-1.39	p=0.0011
Birth length SDS	**-3.09**	-4.72	-1.20	**-2.48**	-3.72	-1.63	p=0.0178
gestational age (wks)	**38.0**	34.0	40.0	**39.0**	33.8	40.0	ns p=0.71
Father's ht SDS	**-1.13**	-2.70	0.45	**-1.35**	-2.55	-0.15	p=0.0446
Mother's ht SDS	**-0.98**	-2.75	0.29	**-1.35**	-2.80	-0.10	ns p=0.12
MPH SDS	**-1.10**	-2.15	0.03	**-1.40**	-2.25	-0.52	p=0.0232

**Table:** 

B: Syndromic versus non-syndromic SGA patients: Anthropometric parameters and pubertal status before start of rhGH therapy							
	Syndromic SGA			Non-Syndromic SGA			Comparison of the 2 groups
	n = 42			n = 230			
	median or n (%)	P10	P90	median or n (%)	P10	P90	p
At start GH							
Age yrs	**7.43**	4.30	12.37	**10.21**	5.43	14.03	p=0.0005
In puberty, n (%)	**4 (9.5%)**			**59 (25.6%)**			p=0.023
Height SDS	**-3.39**	-5.60	-2.62	**-3.07**	-3.74	-2.62	p=0.0253
Height SDS minus MPH SDS	**-2.34**	-4.45	-1.38	**-1.74**	-2.85	-0.83	p<0.0001
weight SDS	**-4.06**	-6.42	-2.13	**-2.91**	-4.38	-1.89	p<0.0001
BMI SDS	**-1.70**	-3.67	0.04	**-1.14**	-2.47	0.27	p=0.0054

ns = not significant.

While mother’s height was not significantly different, fathers of non-syndromic patients were shorter (-1.35 versus -1.13 SDS, p=0.0446) as was MPH (-1.4 versus -1.1 SDS, p=0.0232) (**Table 2A**).

Syndromic SGA patients were started on rhGH therapy at a younger age (7.43 versus 10.21 years, p=0.0005). At start of GH therapy, syndromic patients were shorter (height SDS -3.39 versus -3.07, p=0.0253), especially when taking into account their MPH (Height SDS − MPH SDS: -2.34 versus -1.74, p<0.0001). Syndromic SGA patients were lighter (weight SDS: -4.06 versus -2.91, p<0.0001) and had a lower BMI SDS (-1.70 versus -1.14, p=0.0054) at start of therapy (**Table 2B**).

### 3.3 Syndromic versus non-syndromic SGA patients: Comparison of response to rhGH

After one year of therapy, syndromic patients remained shorter than non-syndromic SGA patients (height SDS -3.04 versus -2.51, p=0.0286; **Table 3**). The percentage of non-responders (delta-height SDS <0.3 after one year of therapy) was comparable (19% in syndromic versus 17% in non-syndromic patients, p=0.69; **Table 3**).

**Table 3:** Syndromic versus non-syndromic SGA patients: Comparison of response to rhGH after the first year of therapy and at onset of puberty.

	Syndromic SGA			Non-syndromic SGA			Comparison of the 2 groups
	n = 42			n=230			
	median or n (%)	P10	P90	median or n (%)	P10	P90	p
After 1 year GH							
Age yrs	**8.60**	5.33	13.30	**11.24**	6.45	15.04	p=0.0006
Height SDS	**-3.04**	-5.04	-1.85	**-2.51**	-3.24	-1.93	p=0.0286
Height SDS minus MPH SDS	**-1.79**	-3.91	-0.89	**-1.22**	-2.30	-0.25	p<0.0001
Weight SDS	**-3.38**	-5.65	-1.46	**-2.37**	-3.54	-1.27	p=0.0001
BMI SDS	**-1.62**	-3.13	-0.02	**-1.03**	-2.20	0.20	p=0.0033
Delta ht SDS 1st yr	**0.54**	0.24	0.94	**0.56**	0.26	0.92	ns p=0.94
Delta ht SDS 1st yr>0.5, n (%)	**22 (52%)**			**134 (58%)**			ns p=0.48
Delta ht SDS 1st yr>0.3, n (%)	**34 (81%)**			**192 (83%)**			ns p=0.69
At start of puberty							
Age yrs	**12.56**	10.86	13.98	**12.39**	10.41	14.30	ns p=0.66
Age yrs in males	**13.03**	11.45	14.86	**13.09**	11.64	14.57	ns p=0.68
Age yrs in females	**11.41**	10.35	12.56	**11.55**	9.77	13.56	ns p=0.75
Height SDS	**-2.35**	-4.01	-0.99	**-2.46**	-3.47	-1.48	ns p=0.59
Height SDS minus MPH SDS	**-1.43**	-2.86	0.32	**-1.06**	-2.31	0.13	ns p=0.081
Weight SDS	**-2.21**	-3.58	-0.83	**-2.22**	-3.34	-0.92	ns p=0.49
BMI SDS	**-1.01**	-3.13	-0.12	**-1.11**	-2.33	0.36	ns p=0.38
Delta ht SDS before puberty*	**1.26**	0.27	2.50	**0.83**	0.16	1.75	p=0.0048

*Only including prepubertal patients under rhGH therapy.ns = not significant.

**Figure 2:**
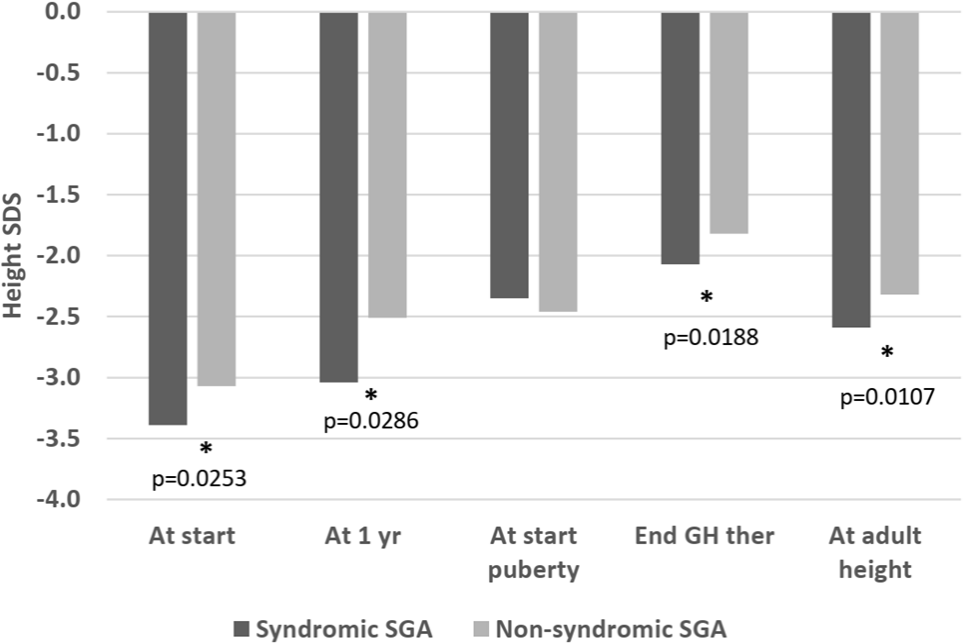
Height SDS, syndromic versus non-syndromic SGA patients: Differences in Height SDS between syndromic and non-syndromic patients before, throughout and after rhGH therapy. * statistically significant.

At the beginning of puberty, there was no longer a significant difference in height SDS between the two groups (-2.35 versus -2.46, p=0.59). Age at start of puberty was comparable (12.56 versus 12.39 years, p=0.66; **Table 3**). However, at the end of therapy, syndromic patients remained shorter (-2.07 versus -1.82 SDS, p=0.0188) and this difference was exacerbated at AH SDS (-2.59 versus -2.32, p=0.0107; **Figure 2** and **Table 4**).

**Table 4:** Syndromic versus non-syndromic SGA patients: Comparison of response to rhGH at the end of therapy and at AH, and details on applied rhGH therapy.

	Group 1: Syndromic			Group 2: Non-syndromic			Comparison of the 2 groups
	n=42			n=230			
	median or n (%)	P10	P90	median or n (%)	P10	P90	p
At end of GH therapy							
Age yrs	**16.01**	14.43	17.83	**16.16**	13.78	18.00	ns p=0.90
Height SDS	**-2.07**	-4.59	-0.87	**-1.82**	-2.77	-0.93	p=0.0188
Height gain SDS	**1.30**	0.35	2.04	**1.33**	0.57	2.05	ns p=0.99
At near adult height							
Age yrs	**17.52**	15.29	20.75	**17.32**	14.95	22.77	ns p=0.85
Height SDS for CA	**-2.18**	-4.95	-1.43	**-1.98**	-2.94	-1.07	p=0.0173
Height SDS 21 yr	**-2.59**	-4.99	-1.57	**-2.32**	-3.30	-1.20	p=0.0107
Height. cm male	**165.5**	150.2	170.6	**165.5**	158.0	173.8	ns p=0.0812
Height. cm female	**148.3**	132.0	155.2	**152.3**	147.2	158.3	p=0.0291
Height SDS 21 yr <-2, n (%)	**30 (71%)**			**144 (63%)**			ns p=0.27
Height SDS minus MPH	**-1.24**	-3.59	0.27	**-0.52**	-1.78	0.49	p<0.0001
Ht SDS 21 yr minus MPH	**-1.70**	-3.79	-0.02	**-0.78**	-2.17	0.12	p<0.0001
Height (cm) in MPH range n (%)	**16 (42%)**			**173 (79%)**			p<0.0001
Total height gain SDS 21 yr	**0.76**	-0.70	1.48	**0.86**	-0.12	1.86	ns p=0.41
Total height gain SDS >1, n (%)	**25 (60%)**			**143 (62%)**			ns p=0.75
Total height gain SDS 21 yr >1, n (%)	**16 (38%)**			**99 (43%)**			ns p=0.55
Total pubertal height gain SDS	**-0.28**	-1.14	1.11	**0.44**	-0.55	1.48	p=0.0001
Duration GH	**8.35**	4.10	11.20	**5.50**	3.10	9.75	p<0.0001
Treatmt interruption, n (%)	**5 (12%)**			**14 (6%)**			ns p=0.17
Total interruption time yr	**3.00**	1.00	3.10	**1.80**	1.00	2.90	ns p=0.10
Mean dose mg/kg day	**0.047**	0.039	0.064	**0.043**	0.035	0.056	p=0.0042

ns = not significant.

BMI SDS of syndromic patients remained lower after one year of rhGH therapy (-1.62 versus -1.03, p=0.0033), but was not different at other time points. Median BMI remained always below P50 throughout the follow-up for both groups (**Tables 3**, **4**).

The age at the end of therapy was comparable in both groups (16.01 versus 16.16 years, p=0.90). Hence mean duration of the GH therapy was longer in the syndromic group (8.35 versus 5.5 years, p<0.0001). The syndromic group was treated with a higher median dose (47 mcg/kg/day versus 43 mcg/kg/day, p=0.0042) (**Table 4**).

As some patients were treated in the setting of studies before the EMA approval of SGA as an indication for rhGH therapy, some patients were treated with a discontinuous rhGH regime. After their study participation, rhGH was interrupted until they could be treated in a medical need program or eventually under the approved SGA indication. This was the fact for 12% of the syndromic patients and 6% of the non-syndromic patients. Median interruption time of their rhGH therapy was 3 and 1.8 years respectively. These differences were not statistically significant (**Table 4**).

**Figure 3:**
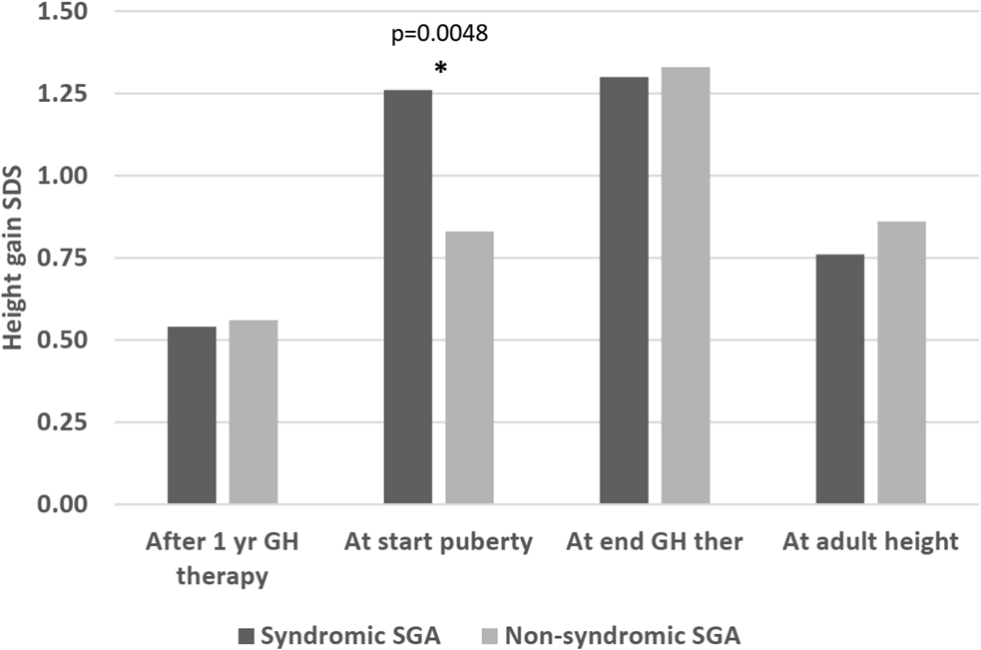
Height gain (delta height SDS compared to start of therapy) in syndromic versus non-syndromic SGA patients: Differences in height gain between syndromic and non-syndromic patients before, throughout and after rhGH therapy. * statistically significant.

Regarding height gain, there was no significant difference either after 1 year of therapy (delta height SDS: 0.54 versus 0.56, p=0.94), at the end of therapy (height SDS 1.3 versus 1.33 SDS, p=0.99), or at AH (height SDS 0.76 versus 0.86, p=0.41). However, before the start of puberty, the syndromic group had a greater height gain compared to the non-syndromic group (height SDS gain 1.26 versus 0.83, p=0.0048) (**Figure 3**). The pubertal height gain was hence lower in the syndromic group (-0.28 versus 0.44 SDS, p=0.0001; **Table 4**).

The majority of patients in both groups remained short (<-2 SDS) at AH (71% versus 63%, p=0.27). The syndromic SGA group remained slightly shorter than the non-syndromic group (height SDS at 21 years -2.59 SDS versus -2.32, p=0.0107), but when comparing final heights for males and females separately, there was no significant difference in males (median final height: 165.5 cm for syndromic and non-syndromic males). Non-syndromic females were taller than syndromic SGA females at final height (median final height: 148.3 cm for syndromic and 152.3 for non-syndromic females, p=0.0291; **Table 4**).

More than 75% of non-syndromic patients reached an AH in their target height range (79%). In the syndromic group, only 42% reached an AH in their target height range (p=<0.0001; **Table 4**).

## 4 Discussion

Our cohort is so far the largest SGA cohort with published information about AH.

The absolute AH we report, is shorter than in some cohorts (4, 5), but similar to some other reports (15) (see **Table 5**).

**Table 5:** Overview of published SGA cohorts with documented AH after rhGH therapy.

Publications	Number of SGA patients	Mean/Median adult height [cm]	Mean/Median adult height [SDS*]	Mean/Median MPH [SDS]	Mean/Median height gain [SDS*]	Inclusion/ exclusion of syndromic patients	Exclusion of patients who started rhGH after onset of puberty
Coutant et al. (27)	70		*mean* -2.0	*mean* -0.8	*mean* +1.0	exclusion	no
Van Pareren et al. (4)	54	*mean* ♀160.1 ♂169.3	*mean* -1.1 (33 mcg/ kg/d)	*mean* -0.9	*mean* +1.8	inclusion of SRS, other syndromic patients excluded	yes
Carel et al. (6)	102	*mean* ♀151 ♂162	*mean* -2.1	*mean* -1.2	*mean* +1.1	inclusion	prepubertal or early pubertal stage included
Dahlgren et al. (5)	77	*mean* ♀159 ♂172	*mean* -1.2	*mean* -1.2	*mean* +1.3	exclusion, except 1 SRS patient	yes
Ranke et al. (15)	161	*median* ♀148.5 ♂161.9	*median* -2.2	*median* -0.8	*median* +1.4	inclusion (55 SRS patients)	yes (min. 2 years prepubertal rhGH)
Renes et al. (14)	136	*mean* ♀159 ♂171.6	*median* ♀-1.9 ♂-1.8	*median* -0.6	*median* +1.1	exclusion	Prepubertal or early pubertal stage included
Beisti Ortego et al. (11)	80		*mean* -1.63	*mean* -1.41	*mean* +0.96	exclusion	no
Becker et al., 2023	272	*median* ♀152 ♂165.5	*median* ♀-2.07 ♂-1.95	*median* -1.36	*median* +1.13	Inclusion, analysis in separated groups	no

*SDS based on chronological age.♀ = female; ♂ = male.

This holds true as well for AH SDS (4, 5, 14, 28). As we are applying SDS for the age of 21 years to calculate AH SDS, we are probably underestimating AH SDS. Other studies calculate AH SDS on the basis of chronological age at final height and hence tend to overestimate AH. But even if we are applying chronological SDS for AH in our study cohort, our AH SDS are below some published data (4, 5, 11, 28), but similar to other reports (6, 15, 27) (see **Table 5**). As syndromic patients reach a shorter AH in our cohort and as they were excluded from several studies (11, 14), this might contribute to our lower reported AH. Several other studies included only prepubertal SGA patients (4, 5, 28), while others reported a better rhGH response when therapy was started before puberty (11, 14). Our cohort comprises quite an important proportion of patients who started rhGH during puberty (9.5% syndromic and 25.6% non-syndromic SGA), this could again contribute to our lower reported AH. On the other hand, in our study, although the syndromic patients were younger at rhGH treatment start, they did note reach a better AH, so further studies are needed to elucidate the effect of timing of the rhGH treatment start.

Furthermore, some patients in our study cohort were treated with a discontinuous rhGH regimen as they had been included in clinical trials before official EMA approval of SGA as an indication for rhGH therapy, then stopped rhGH at the end of the trial and reinitiated it in a medical need program or eventually after EMA approval. This might have compromised AH in our cohort, although de Zegher et al. have shown, that discontinuous rhGH regimens are equally effective, if a higher rhGH is used (29) (this was the case in the study setting before EMA approval).

The majority of our cohort remained short. More than 75% of the non-syndromic patients, but only 42% of the syndromic patients reached a height within their target height range. This might be due to the fact, that MPH was significantly shorter in the non-syndromic group. The difference to MPH (AH SDS – MPH SDS) of the non-syndromic group, if applying AH for chronological age rather than for 21 years of age (in order to compare our results to other publications), is very similar to most published results (14, 15, 28) and to the meta-analysis published by Maiorana et al. (30). This underlines the importance of a careful evaluation of published results regarding rhGH treatment outcome in SGA cohorts (inclusion or not of growth hormone deficient patients, syndromic patients and patients suffering from bone dysplasia, the number of included patients, MPH) as well as the expression of outcome (AH SDS based on chronological age or on SDS for 21 years) in order to interpret correctly the effect of rhGH therapy. **Table 5** provides an overview of the results of the so far published SGA cohorts with documented AH and their inclusion criteria.

However, in terms of height gain, our study showed that no significant difference in total height gain was observed between syndromic and non-syndromic SGA patients. Syndromic patients were more severe SGA, were shorter and lighter before growth hormone therapy, and ended up shorter after growth hormone therapy, but the height gain was comparable.

This contrasts with the results of Adler et al, who describe in a multivariant analysis a worse response to growth hormone in their syndromic SGA subcohort. This study included a significant number of SGA patients suffering from a bone dysplasia in their syndromic subgroup, which might have caused the lower height gain (12).

In our study, the height gain after one year was equivalent in both groups, as was the percentage of non-responders. The percentage of syndromic SGA patients with a delta height gain of more than 0.3 SDS during the first year (81%) was comparable to published results regarding syndromic SGA (16).

However, the growth pattern was different in the two groups. Following a comparable height gain in the first year of therapy, syndromic patients grew better before puberty. Height and age at start of puberty were comparable in syndromic and non-syndromic patients. So, as syndromic patients started rhGH treatment at a younger age than non-syndromic SGA patients, they already had a longer treatment period before reaching puberty, which might have resulted in the greater prepubertal height gain.

The pubertal height gain of syndromic patients was lower, thus they ended up shorter than non-syndromic patients. This could be due to the fact, that SRS patients accounted for 15% of the syndromic patients and that SRS patients present an earlier pubertal onset (18) and an earlier adrenarche than other SGA patients (31). SRS boys with an early adrenarche are known to be taller at gonadarche but to end up as short as the boys with normal adrenarche (32). However, in our cohort, age at start of puberty was not younger in syndromic patients compared to the non-syndromic ones. We lack data on adrenarche in our cohort.

Concerning adult height, if applying the chronological SDS for adult height, the AH SDS outcome of our syndromic group corresponds to most published AH SDS of SRS patients treated with rhGH (19, 32).

For the second largest group of patients (FAS patients) in the syndromic group, no data regarding AH after rhGH and timing of puberty in a larger cohort have been published. Sparse data (based on seven patients) are available on rhGH response in the first two years indicating a worse rhGH response than observed in SRS patients (16).

SGA patients were treated with a higher rhGH dose than the EMA- approved dose. This is due to the fact, that some patients have been treated with higher rhGH doses in the setting of clinical trials preceding EMA approval. An analysis of adult height in large SGA cohorts including only patients started on a rhGH therapy after 2003 in order to evaluate the effect of the currently applied dose recommendation has not yet been published.

Our syndromic group comprised 17 patients (40%) with no defined syndrome. In 47% of these patients a genetic analysis has not been contributory. Of note, in the majority of patients no update of the genetic analysis was carried out and most patients only had a karyotype and/or a SNP analysis performed at the time of their rhGH treatment start. As the genetic field is developing fast and more and more genetic disorders are unraveled (33, 34), it is quite possible, that a molecular genetic analysis performed today in these patients would substantially decrease the number of undefined syndromes. In 9 syndromic patients, no genetic analysis has been performed. This might be due to the fact, that our cohort comprises patients who were treated more than 20 years ago, when genetic analysis was not that widely and easily available or as the patients completed the SGA criteria and had access to rhGH therapy, a further diagnostic work-up might have not been regarded as indispensable.

Another weakness of our study is, that although we have established and applied criteria to divide the patients into the syndromic and non-syndromic group, it is not excluded, that there might still be some syndromic patients in the non-syndromic group. Some syndromes have only very subtle clinical signs which might be overlooked. Further, as this study is a retrospective study based on a registry, if symptoms have not been documented in our database, patients might have been falsely classified as non-syndromic.

In conclusion, we report, that syndromic SGA patients have a similar height gain after rhGH therapy, as non-syndromic SGA patients. Hence, syndromic SGA patients should not be excluded from a rhGH therapy, nor do they have to be excluded from a SGA cohort analysis of rhGH response. Syndromic patients were significantly shorter before rhGH therapy and remained significantly shorter in stature after rhGH therapy. An AH in the normal range was achieved only in ca. 1/3 of all patients, but 73% reached an AH within their target height range.

## Data availability statement

The raw data supporting the conclusions of this article will be made available by the authors, without undue reservation.

## Ethics statement

The studies involving human participants were reviewed and approved by Comissie Medische Ethiek, VUB. Written informed consent to participate in this study was provided by the participants’ legal guardian/next of kin.

## Author contributions

MB, MT and DB designed the study. MB, MT, JDS and DB contributed to the data collection. MT contributed to the data analysis. MB wrote the manuscript. All authors contributed to the article and approved the submitted version.

## Members of the BESPEED group

Anne Rochtus^2,3^, Anne-Simone Parent^6^, Daniel Klink^4^, Guy Massa^7^, Karl Logghe^1^, Kathleen De Waele^5^, Kristina Casteels^2,3^, Martine Cools^5^ and Willem Staels^8^

^1^ AZdelta Hospital, Roeselare, Belgium

^2^ University Hospitals Leuven, Leuven, Belgium

^3^ KU Leuven, Leuven, Belgium

^4^ ZNA Queen Paola Children´s Hospital Antwerp, Antwerp, Belgium

^5^ Ghent University Hospital, Ghent

^6^ University Hospital Liège, Liège, Belgium

^7^ Jessa Hospital, Hasselt, Belgium

^8^ University Hospital Brussels, Brussels, Belgium
